# Baptist church and empowerment of women and girls with disabilities in Mhondoro-Ngezi

**DOI:** 10.4102/ajod.v15i0.1661

**Published:** 2026-06-13

**Authors:** Grinwell Mugoni, Macloud Sipeyiye

**Affiliations:** 1 Department of Pastoral Ministry, Baptist Church, Gweru, Zimbabwe; 2 Department of Religious Studies and Ethics, Faculty of Arts and Humanities, Midlands State University, Zvishavane, Zimbabwe; 3 Research Institute for Theology and Religion, College of Human Sciences, University of South Africa, Pretoria, South Africa

**Keywords:** disability, discrimination, feminist disability theology, Judeo Christianity, poverty, spiritual capital, stigmatisation, spiritual capital

## Abstract

**Background:**

The Judeo-Christian and Shona indigenous religious spaces often reinforce the disempowerment of people with disabilities in general and women and girls in particular. In Zimbabwe, women experience the double vulnerability of being a woman in a patriarchal society and living with disabilities. Disability may increase poverty, while poverty may delay health-seeking behaviours and expose people to health risks that culminate in disability.

**Objectives:**

The article explores how the Ngezi Baptist Church is re-appropriating its theology of disability to mitigate the stigmatisation, exclusion and discrimination of women and girls with disabilities, thereby reducing poverty among them.

**Method:**

It employs the feminist disability theology in reshaping disability perceptions in Zimbabwe. Research data were obtained through focus group discussions and in-depth interviews with purposively selected participants.

**Results:**

Local churches have great potential to reinterpret their doctrines and harness their spiritual capital to initiate inclusive social and economic initiatives aimed at improving the well-being of women and girls with disabilities.

**Conclusion:**

Ngezi Baptist Church is embracing the feminist disability theology that conceives of God as contextual. It encourages reimagining God in various forms of human variation in its drive to unlock the potential of the vulnerable categories. It inspires the able-bodied and the differently abled persons to work together towards alleviating poverty.

**Contribution:**

The research provides a unique feminist disability theological analysis of the contribution of a local church towards reshaping community perceptions about women with disabilities and empowering them through upholding their dignity and reducing poverty among them.

## Introduction

### Background

Churches have a critical role in the development of society. One developmental area where churches are expected to play a proactive role is in empowering persons with disabilities in general and women with disabilities and girls with disabilities in particular. Amanze (2019) asserts that the church, as an agent of change, can mainstream disability challenges holistically leveraging on its spiritual capital. A sizeable population of the world, estimated at 16%, is said to live with disability, with 80% living in the Global South (WHO 2023). According to Chadambuka et al. (2024), the Zimbabwe National Statistics Agency (ZIMSTAT) and the United Nations Population Fund (UNFP) put the national disability rate in 2017 at 9%, with a superior number of women than men. The Judeo-Christian and Shona indigenous religious spaces have often reinforced the disempowerment of persons with disabilities. The coalescing of the two traditions in a single geographical and cultural context poses a great threat to the said categories of people. Women and girls are the most affected, as they experience the double vulnerability of being a woman in a predominantly patriarchal religio-cultural context and living with disabilities. There is an intersectionality of poverty and disability in that, where the latter may increase the former, poverty may cause delays in seeking medical assistance and expose people to health risks that culminate in disability (Moodley & Grayham 2015). There is no uniform treatment of disability in the religious landscape of Zimbabwe (Sande 2023). Various Christian denominations have varying theological and ideological positions on persons with disabilities. The African indigenous religion is another contending spiritual option that forms the base with its unique but often coalescing position on the same. The various religious positions reinforce some already existing cultural perceptions about the notion of the wholeness of the body and patriarchal traditions that despise the female gender. To this end, it is a challenge for churches to speak with one voice against barriers to a fulfilling life for persons with disabilities. This diversity calls for a critical contextualisation of the discourse of disability, hence the choice of the Ngezi Baptist Church. The article uses the feminist disability theology framework to analyse the Judeo-Christian and Shona indigenous perceptions of disability to give a concrete context within which we can explore the various strategies that Ngezi Baptist Church utilises or can utilise to bring to the centre the discourse of disability with the intent to liberate and/or empower women and girls with disabilities from stereotyping and its causative challenges that include poverty. The article, informed by the data from the Ngezi Baptist Church’s engagement with disability, concludes that religion in general and the church in particular, has the potential to transform perceptions of communities about disability. Ministers of religion and other religious practitioners are open to embracing initiatives bent on capacitating them even further with skills to reinterpret their theologies to provide inclusive spaces that promote justice, especially fighting discrimination, exclusion and stigmatisation for persons with disabilities.

### Theoretical framework

The proponents of the feminist disability theology include Black (1996) and Eiesland (1998, 2002), whose thrust of the theology celebrates the ‘identity and dignity of women’s differently-abled bodies’ (Chisale 2020:2). The feminist disability theology challenges patriarchy and the accompanying social prejudices often targeting persons with disabilities. Eiesland (1998) proposes that God should be reimagined in multiple and varied forms of the human body. Schumm (2010) supports this position by reconstructing the concept of disability through a dual prism that is feminist and theological in perspective. The theory addresses how women with disabilities relate and converse with the world around them and God in the essence of being equal creations to God’s image, where women with disabilities have the opportunity to bring their lived experiences that include their sexuality in relation to the Almighty (Chisale 2020). The feminist disability theology is liberative in nature. It seeks a paradigm shift from the stereotyped hermeneutic approach and societal perceptions towards persons with disabilities. It is against this backdrop that African women theologians realised that women with disabilities are not different from oppressed people because their conceptualisation of God is heavily influenced by their lived experiences. Christianity, influenced by the Hebrew Bible and African religio-cultures, views disabilities as curses and a manifestation of witchcraft (Chisale 2020a). The theory aims at inspiring the transformation of the church, society, government and policy formulation institutions to promulgate relevant policies, laws, rules and programmes that advocate the dignity and rights of persons with disabilities. This will enable them to gain equal and unhindered access to social and economic amenities and all other requisite aspects of life (Ndlovu 2016). The feminist disability theology vouches for the reconstruction of disability that shuns linking disability with sin. It is a theology that inspires efforts that are meant to assist persons with disabilities to realise their capabilities and creativity with the purpose of eliminating stigmatisation and marginalisation (Mutswanga, Makoni & Chivasa 2015). The theology motivates the enactment of laws, policies, rules and programmes that affirm and promote the dignity and rights of of persons with disabilities, including their access to economic, spiritual and social (Ndlovu 2016).

## Research methods and design

The goal of the research was to observe the participants in their natural setting and document their observations rather than testing previously formed hypotheses. The study employed the qualitative research methodology to have access to the lived experiences of women with disabilities and girls with disabilities in their natural setting. Qualitative research methodology is primarily an exploratory research approach employed to understand the meaning that the subjects of the research attach to their experiences of phenomena (Willig 2001). Data obtainable are presented as descriptive text production than numerical outputs. Resultantly, the researcher is the main tool of analysis (Sanjari et al. 2014). The researcher collects open-ended emerging data with the primary intent to develop themes from them (Creswell 2007). So, we analysed data through thematic coding of the excerpts from the interviews with respondents.

### Sampling techniques

The researchers employed focus group discussions and in-depth interviews to gather data on the experiences of the women with disabilities and girls with disabilities in wards 1, 14, and 16 of Mhondoro-Ngezi. Participants were purposively selected on the basis of the researchers’ knowledge of the population to provide the best information to address the research problem. Five adult women with disabilities aged between 35 years and 65 years, five girls with disabilities aged between 13 years and 17 years, and three men with disabilities were sampled in each of the three participating groups. The guardians of the girls gave consent and went on to give interviews on their behalf for the sole reason that legally and ethically minors cannot give consent in Zimbabwe. A total of three focus groups were conducted in each of the three participating wards with five women, five girls, and three men each. One of the three focus group discussions in each of the three wards was conducted with women, girls, and men without disabilities to get their views and opinions about the experiences of women with disabilities and girls with disabilities. As Eiesland (1994) observes, persons without disabilities have a role to play in untangling existing barriers to pave the way for persons with disabilities. Two unstructured interviews with two women with disabilities and two girls with disabilities were also carried out in each of the three wards to get their experiences, which we used to determine the extent to which their conditions affect them daily. While the focus of the research was primarily on women and girls, we considered it imperative to get the views of men with and without disabilities on the experiences of women and girls with disabilities. We felt that these categories had important information about the experiences of the women and girls with disabilities, as some of the social injustices they experienced were perpetrated by men. The subject of disability is a sensitive area among the Shona. The persons with disabilities are often treated with secrecy to the extent that some of them may not venture into public spaces for the rest of their lives. They constitute the ‘hidden populations’. This meant that some potential participants would not be easily accessible to the researchers. The researchers utilised snowballing as a supplementary sampling technique. Ngezi Baptist pastors were interviewed for their views on engaging the church in transforming the lives of the women with disabilities and girls with disabilities in view of the intersectionality of disability and poverty.

### Disability

The United Nations Convention on the Rights of Persons with Disabilities (United Nations 2006) considers persons with disabilities to include:

[*T*]hose who have long-term physical, mental, intellectual or sensory impairments which in interaction with various barriers may hinder their full and effective participation in society on an equal basis with others. (p. 3)

It is important, therefore, to note that different knowledge bases, cultures, contexts, values and religious beliefs have a bearing on disability. Thus, disability is perceived differently and as such, there are various models of conceptualising it that include the cultural, social, human rights model and many others. The cultural model of disability is crucial because the functioning of persons with disabilities depends on culture (Sande 2023). Cultural beliefs and attitudes pose as powerful instruments towards how society views persons with disabilities. Notwithstanding the values obtainable in culture and its dynamic nature, the model, looks at the cultural impediments which act negatively on persons with disabilities (Snyder & Mitchell 2006). Culture informs the boundaries and provides attitudes that define the perceptions people hold about a person with disabilities. Cultural values often have a bias towards the male gender, suffocating the women and girls who suffer the ‘double trouble’ of being a woman and living with disabilities. Culture enjoys an unholy covenant with religion, where the latter reinforces the existing cultural perceptions about the notion of the wholeness of the body and traditions that despise the female gender, underscoring the intersections between disability, gender and religion. It is therefore important to reflect on the Judeo-Christian and African indigenous traditions on disability.

### The Judeo-Christian traditions and disability

Religious beliefs have a strong influence on perceptions and attitudes on how persons with disabilities view themselves, and how others and the rest of the world perceive them (Ahmad 2015). Judaism and Christianity are not very supportive of persons with disabilities, yet they ‘influence perceptions, attitudes and remedial and coping practices towards disability by both caregivers and the community members’ (Ahmad 2015). Many people fear negating religion and its teachings because they are fearful of the unknown consequences. Christianity is one of the dominant religions in Zimbabwe, which is believed to enjoy an estimated 87.4% of the total population (Mutswanga et al. 2015). Christians often link disability to sin. This derives from the moral or religious model of disability that attempts to explain disability from the ancient Greek and Judeo-Christian perspectives (Sande 2023). The model conceives of disability as punishment from the supernatural for the people’s wrongdoing. The model blames the family as responsible for causing disability, leading to the discrimination and stigmatisation of families with members with disabilities from participating in society (Rimmerman 2013). As Boaz (2015) observes, persons with disabilities are presumed to go to church only for healing, and this is because of the connection of faith and prayer, with claims of healing which proclaim that the blind will see and the deaf will be able to hear. However, when no ‘healing’ occurs, a person with disabilities is then labelled as a seasoned sinner. Judaism and Christianity often employ disability figuratively and metaphorically to strengthen and bolster their views (Ojok & Musenze 2019). Examples include Exodus 4:11 and Isaiah 56:10. When religion uses language and vocabulary, in the figurative or imagined sense, in direct reference to stigmatised groups, this notion exemplifies discrimination and social stigma (Ojok & Musenze 2019).

### The Shona indigenous thought on disability

The Shona indigenous thought has its own conceptions that shape the lived experiences of persons with disabilities in Zimbabwe (Sande 2023). The Shona hold ambivalent perceptions about persons with disabilities, where on the one hand, they are abusive, stigmatising, denigrating and exclusionary, while on the other hand, they are supportive and inclusive, thus discouraging their abuse and ill-treatment. These varying attitudes are expressed in the proverbs, folktales and taboos of the Shona’s oral tradition which either embrace or look at disability with disdain (Makamure 2017). For example, the Shona proverb, *seke urema wafa* [laugh at disability only when you are dead] is indicative of the Shona’s distaste of stigmatising disability. Thus, the Shona indigenous thought is not through and through condemning of disability but it also provides ways of coping, managing and curbing the challenges that affect persons with disabilities. The religious, cultural and social values, perceptions and attitudes play a pivotal role in either constructing and legitimising disability or deconstructing disability and integrating it.

### Causes of disability among the Shona of Mhondoro-Ngezi

There are various causes of disability among the Shona of Mhondoro-Ngezi. The focus group discussion that was constituted of women without disabilities held in Ward 1 brought out the view that disability comes in different types and forms and at different stages in life. These forms include but not limited to impairment of vision, speech impediment, dumbness, deafness (or both), paralysis, mental illness and albinism. Participants in the group reported that some people are born with disabilities that are often attributed to the work of evil spirits, though some people conceive of them as the will of the creator. Belief in evil spirits is prevalent among the Shona. Witches are a feared source of evil known to have extraordinary powers to hurt and afflict other people. Thus, the sources of disabilities make the Shona fear the persons with disabilities (Sande 2023). Any form of disability is associated with misfortunes that are triggered by evil spirits. One woman participant without disability reported that disability is also caused by accidents, illness or old age. Mashiri (2000) concurs that accidents are associated with angry ancestral spirits or alien spirit. Another woman participant without disability averred that disability is a result of fortune-enhancing rituals. She cited cases of disabilities, especially mental illness, that are believed to be caused by charms that are often acquired by businesspeople to boost business. It may either be a result of inappropriate use of the charms or that the charms work by affecting disability in some members of the family of the person who acquired them. Humbe (2024) posits that:

[*I*]n some cases, business rituals performed by certain families result in girl children becoming mentally ill [*and*] … once the girl-child becomes mentally ill, the biological fathers then sleeps with her to ensure success in their family business. (p. 7)

All these cases above serve to show that disability manifests in different ways among the Shona, and it is conceived differently within different cultures.

### Ethical considerations

Ethical clearance to conduct this study was obtained from the Midlands State University (MSU) Department of Religious Studies and Ethics Research Ethics Committee, and ethics consent was received on 18 December 2022.

The authors ensured that all necessary steps were taken to avoid violating the rights of the participants. Verbal informed consent was attained from the participants, who committed to voluntary participation; establishing good rapport, confidentiality and anonymity. The authors also reiterated the participants’ right to opt out of the study at any stage of the research or to decline to respond to certain parts of questions with which they felt uncomfortable. The study used pseudonyms and alphanumeric acronyms to protect the identity of all participants. For example, NBCP1 represents Ngezi Baptist Church Pastor one and WMU2 for Women Missionary Union participant member two. Woman with disability and women with disabilities are written in full to avoid labelling these women.

## Results and discussion

### Experiences of women and girls in Mhondoro-Ngezi

The focus group discussion with disabled women shared that women and girls with disabilities are subjected to stigmatisation, discrimination and exclusion even by their biological families owing to their condition. One participant narrated how a disabled girl was literally hidden in the house by her family. The only time she went outside was when she had to use the communal toilets outside:

[*T*]hey keep her inside the house on the pretext that she walks too much, and is most likely to get lost if she is allowed to go outside the house. (Woman without disability 1, 60 years old, unemployed)

The relatives always claim that she is possessed and that unknown spirits are calling her. As a result, she would not go outside or else she will go astray because of the demonic spirits dwelling in her. The perceptions of the relatives can be argued to be influenced by the Judeo-Christian and African indigenous perspectives on disability.

A visually impaired woman narrated that she lost her sight after a snake spat into her eyes. She failed to get immediate medical attention. She could not work on the farm, so she later moved to the city in search of economic opportunities. However, in her search for love, belonging and companionship, she has been repeatedly raped and sexually abused with false promises of money and marriage. Sexual abusers of women and girls with disabilities often commit such heinous crimes under the misconception that they are doing their victims a favour since they are perceived to be sexually starved (Chadambuka et al. 2024).

One participant in Ward 16 focus group discussion (FGD) one explained the experiences of women with disabilities with public transporters:

‘The public transporters see us as a nuisance. They always say we are taking up too much of their time and delaying them.’ (Woman with disability 1, 60 years old, unemployed)

A woman participant with a disabled girl-child had this to say:

‘I have no problem paying fees to get my daughter educated. My biggest challenge is transport to and from school by public transporters. My daughter cannot manage to go to the bus stop and pick-up point where all the kids are picked from on her own. Even if we do accompany her, once in a while, the transporters are unwilling to have our child and help her as they feel she needs a lot of assistance and this interferes with their business operations.’ (Woman with disabled child, 55 years old, entrepreneur)

One participant in the Ward 1 focus group with visual impairment corroborated:

‘The public transporters see us as a nuisance. They always say we are taking up too much of their time and delaying them.’ (Woman with disability 2, 50 years old, unemployed)

The views above express the challenges that the visually impaired girls with disabilities faced in accessing public transport and amenities. Commuting to urban areas was a big challenge for women with disabilities as well, as commuter omnibus operators were reluctant to offer a helping hand, especially in folding wheelchairs and getting the women with disabilities on board and getting them a comfortable seat. The visually impaired women in Ward 1 had problems getting to the communal public transport pick-up points, since in most cases they needed a family or community member to guide them there. Thus, resource constraints and the general marginalisation and invisibility often limit PwDs’ access to social services such as transport because of lack of assistive technology (Lewis 2004).

### Limited educational and employment opportunities

The women and girls with disabilities in the Mhondoro-Ngezi District had very limited educational opportunities. The women had dropped out of school at Grade 7 and started fending for themselves, while girls were struggling to get a place at mainstream schools because the schools did not have support structures for inclusivity. Of all the women participants with disabilities, no one was formally employed; and only one was self-employed as a vendor. One participant said:

‘I did not go beyond Grade 7. My Uncle was very brusque with me and continually reminded me that I was lucky to have set my foot in class.’ (Woman with disability 1, 60 years old, unemployed)

At one school in Mhondoro-Ngezi, the School Head has not been able to offer a visually impaired girl-child a place because the school has neither facilities nor teacher that cater for the visually impaired children:

‘We would want to accommodate all children with disabilities in line with the Zimbabwean Government policy of Inclusive Education; but the question of suitability of our schools is another issue. We do not have classrooms that are properly equipped for children with disabilities, more so the girl-child. What makes the situation worse is the limited teaching materials for children with hearing or visual impairments. The visually impaired child is at the mercy of his peers for assistance, who can either accept or refuse to offer assistance.’ (Man, 62 years old, school head)

The parent could not send his daughter to Jairos Jiri Institution in Rimuka because he could not afford both the day and boarding fees. The transport costs for day schooling were also prohibitive. As a result, the girl-child living with a disability missed an opportunity to go to school and improve her life. Most children with disabilities, especially girl children, are denied access to basic education because they come from very poor families which cannot afford the high fees normally charged by the special needs schools. Ndlovu and Nyoni (2021) say that:

[*I*]n the education sector in Zimbabwe, school fees have risen sharply due to high inflation, and as a result, many families cannot afford to send all of their children to school and much less those children with disabilities. In this regard, girl children and women are more disadvantaged. (p. 45)

Most schools are unable to accommodate children with visual impairments, both in terms of teacher capacity and infrastructure (Ndlovu & Nyoni 2021). Mandipa (2014) observed that Zimbabwe has very few officials trained to handle people with intellectual disability (ID) and with hearing and speech functional disabilities. In most cases, the infrastructure is unfriendly, for example, the toilets cubicles are too high for people with both intellectual and physical disabilities. The PwDs constitute a category that faces conditions that are beyond their control. Thus, they may be viewed as people experiencing social suffering and structural violence that is woven into the societal structures, ideologies and institutions (Pincock et al. 2022).

### Accessing health services

The focus group interviews indicated that PwDs, and especially women with disabilities, faced considerable challenges in accessing health services. Firstly, the three local clinics that serve Ward 1, 14, and 16 are located far from where most of the participating women and girls with disabilities live. They have to cover considerably long distances to get to their respective health centres. Secondly, the local health centres are often insufficiently equipped. They do not have adequate basic medications and special medications required by women and girls with disabilities, for example, skin lotions required by people living with albinism. They also do not have ambulance services for emergency cases. The women and girls with disabilities have to travel long distances to get to the referral centres in the district. Most roads in Mhondoro-Ngezi are untarred, bumpy and uncomfortable. Travelling to the St Michaels Hospital and Kadoma Hospital referral centres, relatively long distances, compounded the women and girls with disabilities’ challenge of accessing crucial social services. Worse, these PwDs often do not meet the required transport costs, and they would normally fail to travel to these referral centres. If they finally do, the few medical specialists are not always available. One participant in one of the FGDs said:

‘Our disabled counterparts, especially women, face a number of problems when they want to go to clinics and the referral hospital which is St Michaels or Kadoma Hospital. Our healthcare facilities are only accessible to those who can afford to pay for transport. Disabled people are suffering terribly. The government must help.’ (Man, 45 years old, local leadership)

Thirdly, the women with disabilities reported about the general negative attitude of the health personnel towards PwDs. They are generally not comfortable with getting in contact with PwDs. The participant in one of the group discussions opines that:

‘Generally, people are not comfortable with getting contact with PwDs, and health staff are not an exception. This may be attributed to some religious and cultural beliefs surrounding PwDs. Disability is often associated with curses in many religious and cultural spaces. So, they may be afraid of contracting the “curse.”’ (Woman without diasbility 2, 38 years old, farmer)

The perceived and real negative attitudes of health workers towards PwDs are a barrier to access to health services and constitute stigma and discrimination. They do not encourage health-seeking behaviours among PwDs.

### Stigma and marginalisation of disability

Women and girls with disabilities are subjected to severe marginalisation and discrimination from their families and society. Failure to live up to the expected standards for womanhood imposed by society leads to a diminished womanhood and low self-esteem in women with disabilities. Owing to their disabled bodies, the immediate family is reluctant to provide them with moral support to engage in normal heterosexual relationships. Instead, they are ascribed with an asexuality identity where their sexual and reproductive health issues are non-existent; they are generally assumed either to be or ought to be asexual (Chadambuka et al. 2024). Most girls with disabilities have grown up without the love of their biological mothers. One participant, a caregiver grandmother living without a disability, stressed that young mothers of children with disabilities often transfer their caregiving roles to their mothers or other close relatives for fear of the religio-culturally driven myths associated with disability. She remarked that:

‘I am staying with my grandchild because the mother ran away. I do not even know where she is right now. At least she did not kill the baby. However, I take care of every need of this child without the help of the mother. She feared the disability and ran away.’ (Elderly woman without disability 3, 65 years old, semi-retired vendor)

These fears are propelled by the moral and religious model of disability that explains it as a punishment from the supernatural for people’s wrongdoing. They are compounded by the medical model’s artificial dual classification of people that makes the society to view PwDs as people who need their bodies to be corrected (Sande 2023). Choguya (2021) postulates that:

[*C*]ommunities play a key role in perpetuating the discrimination and stigma of disability; hence, the fear and shame surrounding disability propels parents to leave their children in solitary thereby segregating them from other children and the wider community. (p. 41)

Such social constructions constitute protective abuse, which is likely to result in sexual violence against women and girls with disabilities and concretise their invisibility in public. The experience of stigma and marginalisation subjects women and girls with disabilities to poverty. The social model of disability proffers a plausible approach to the social phenomenon. The model considers persons without disabilities as having a role to address the existing barriers that suffocate women and girls with disabilities in particular and PwDs in general.

### Ngezi Baptist Church and women and girls with disabilities

Churches play a pivotal role in transforming communities for the better. They are involved in projects that reduce poverty among vulnerable groups in society. Ngezi Baptist Church offers support to women and girls with disabilities in the Ngezi and Mupamombe suburbs. The church often has to contend with financial constraints in the implementation of the projects, where it capitalises on its spiritual capital to mobilise the little available resources for the common good. These church activities are inspired by the disability theology. Creamer (2012:339) describes disability theology as a discipline that deals with endeavours which have been carried out by religious traditions to address the notions of disability and impairment with a view to providing ‘constructive possibilities for inclusive theological work in the future’. The research findings show that Ngezi Baptist Church has operationalised church-driven community development projects which have targeted largely the women and girls with disabilities since 2017. One participant reported that the church leadership has identified women with disabilities and women who are guardians to girls with disabilities and helped set them up in the vegetable vending business. The project aims to empower the local women and girls with disabilities. She had this to say:

‘Ngezi Township and Mupamombe, low-income and high density suburbs, were identified and used as pilot project areas to implement church-driven community development project for women with disabilities over the last five years. Two women with disabilities were identified by the church and given basic training in accounting for small businesses in June 2017. Thereafter, they were given capital to start vegetable vending business from within their premise yards and the shops. Four Church leaders were chosen to oversee the project. Their businesses are self-sustaining to date.’ (Woman Missionary Union 1, 48 years old, teacher)

The Ngezi Baptist Church’s women’s department, WMU, also borrows from the ideals of feminist theology of disability and is encouraging both non-disabled women and girls and women and girls with disabilities to reject their exclusion from the community’s socioeconomic activities. Through their sermons and interaction with women with disabilities, they challenge the fear and shame that surround disability as a social construct. They emphasise the need for women with disabilities to counter-attack this fear by reinventing emancipatory ‘concepts of disability that challenge the notion of the normative body’ (Chisale 2020a). The WMU department has an all-inclusive programme aimed at PwDs in general and women and girls with disabilities in particular that advocates for the inclusion of all women in the fight against the subjugation of the feminine body. It advocates for a departure from naive hermeneutics, especially the Judeo-Christian standpoint on disability and embraces a liberative theology that emphasises on inclusivity because Christianity is founded on the inclusive Christ. One woman without disability, a member of the WMU (WMU 1) said that they reach out to men and implore them to work in solidarity with women because their masculine body is also subject to diminished ability. All human bodies are fragile, and as the human body developmentally progresses, fragilities become evident; and the crucifixion of the body of Christ is a good example of breakability. Chisale (2020a:8) postulates that God is associated with human vulnerability and anguish ‘through the crucifixion and death, resurrection transforms the pain of human suffering into renewed divine hope’.

Women Missionary Union participant member two reported that Ngezi Baptist Church, through the Baptist Men’s Fellowship (BMF) and the WMU, conduct lessons regarding the sexuality of PwDs and women with disabilities when these respective arms of the church meet for their monthly meetings. She said that some of the common themes covered include the teachings that women with disabilities are not different from any other burdened human being, since her views of God culminate from her lived experiences. Her submissions are echoed by Chisale (2020a:1), who refers to women with a disability as having a ‘differently abled body’. The church is, thus, advocating for harmony and solidarity between able-bodied women and women with disabilities, and able-bodied men and men with disabilities. The call for harmony and solidarity is more emphasised for women without disabilities and women with disabilities because, as Chisale (2020a) has observed in other contexts, both categories are sexually affected by purity and morality theology.

One participant shared her excitement from one of the WMU’s training sessions. She quoted the WMU chairperson as having said:

‘Our Biblical God and our traditional Ancestors are a source of life and inclusion. So, whoever is born visually impaired, crippled or has got albinism should be embraced as a blessing irrespective of his disability or ability. There is a person on one hand and a disability on the other, and our personhood comes first and foremost, and whatever disability a person must be embraced as a diversity.’ (Woman without disability 4, 54 years old, entrepreneur)

Another participant explained that the teaching was quite refreshing and encouraging. She has been able to embrace her disability. She remarked that:

‘I no longer have any fear of my disability. This fear that I used to harbour was only destroying my dignity and self-worth.’ (Woman with disability 3, 50 years old, unemployed)

Women and girls with disabilities are often subjected to disrespectful behaviours that expose them to harm. The church has made an impact on the women with disabilities community in Mhondoro-Ngezi. The change in societal attitude is being spearheaded from within the church through its ‘*Engaging Women with Disabilities Sessions in Ngezi and Mupamombe*’. The WMU department uses the African ubuntu principle, asking communities to foster mutual relationships, where the pain of one member should be seen as pain for the whole community.

Ngezi Baptist Church Pastor One retorted that one of the BMF’s mandates is to reach out to the marginalised to provide care in terms of spiritual capital sustenance services inclusive of, but not limited to music, counselling, preaching, teaching through Bible studies, prayers and the use of media (posters, flyers, church-related regalia, and social media) to foster a positive attitude towards PwDs. The BMF encourages its Youth Department to compose music and songs that tell the story of the PwDs in a positive way to facilitate the embracing of the same. Music is a powerful tool that can inculcate and reinforce positive attitudes towards the marginalised. It can go a long way towards transforming attitudes, perceptions and behaviours.

Ngezi Baptist Church Pastor One enunciated that the BMF has, through the pastoral ministry and pastoral care outreach programmes, brought to the fore the themes of the ‘disabled’ God. They use scriptures which show human-likeness to God and an all-inclusive image of God, God’s love and compassionate nature, and the social justice due to PwDs. These themes challenge the conventional theology of the perfect body. Chisale (2020a) concurs when she argues that:

[*N*]ormative of the perfect body is a misnomer because the focus of the flawless body overlooks the fact that people have diverse bodies, and a perfect body image is ambiguous because it is not defined. The image of God is all-encompassing image. (p. 8)

The pastor explained that Ngezi Baptist Church’s pastoral care ministry is about changing mindset from the debilitating effects of patriarchy towards God’s Kingdom; and when the local church carries out its pastoral ministry endeavours, it strives to facilitate liberation, emancipation and empowerment of able-bodied persons and PwDs, and this is done by reinforcing the idea that all human creations are equal before God, despite the physical differences. The pastoral care ministry is guided by the maxim that in the Kingdom of God, everyone is welcome, empowered and liberated by the grace of God; hence, the inclusion of all females and women and girls with disabilities in holy spaces where they are expected to participate in the ministry of God’s Word on earth. This is a radical break from the first, the restriction to the reachability of God, holy spaces, and ministering in the pastoral ministry contexts that is often denied on the grounds of the hermeneutics of the purity laws in the antiquated Judeo-Christian perspectives. Secondly, it is a break with the general misleading assumption that disabled persons grace the holy spaces in search of healing. The Ngezi Baptist Church believes in the doctrine of a God incarnate, an all-inclusive and an accessible God who is available to embrace all, regardless of identity. Ngezi Baptist Church is on a drive to challenge women and men, abled and disabled, to unite in re-interpreting purity and disability since everyone is affected; and women, and mostly women with disabilities, are excluded from participating in the inclusive pastoral care ministry.

### Limitations of the study

The study can be said to be limited in the sense that it deals with a small sample with a specific Christian denomination in a specific cultural context. Its findings may not be representative of all Christian denominations in Zimbabwe, and therefore are not likely to be applicable to relevant broader groups. Generally, the research findings of qualitative research usually falter on their non-generalisability; that is, the findings may not be applicable beyond the sample of a given study (Stausberg & Engler 2011). However, we concur with feminist critics who argue that generalisability and related concepts are often interpreted in universalising and hegemonic terms, failing to recognise the situated nature of truth and/or reality (Stausberg & Engler 2011). We concur with Willis (2007) that conceptualising reality in this way trashes its relativism. People’s perception of reality is always conditioned by their experiences and culture.

### Recommendations

The authors offer the following recommendations:

- The promulgation of new disability-friendly laws and strengthening the implementation of existing laws and policies that include the National Disability Policy with a view to ensuring equal access to social services that include education, healthcare and justice for women and girls with disabilities.
- Ensure the provision of accessible infrastructure that includes accessible public facilities, transportation and reliable communication systems to ensure equal participation across gender lines.
- Roll-out capacity-building programmes on gender and disability rights and inclusive practices for public officials, law enforcement agents and healthcare providers.
- Create religious spaces that promote inclusive practices that include inclusive language, accessible worship spaces and sermons that raise awareness about gender and disability.
- Support efforts that build confidence and economic empowerment for women and girls with disabilities.
- Championing community-based support groups that promote gender and disability awareness and inclusivity.
- Awareness campaigns that challenge life-diminishing cultural norms and gender stereotypes and promote understanding of disability rights.

## Conclusion

The article has noted that societies shape views on disability, and the church has a role in reshaping societal perceptions and constructions on disability. The church has a mandate to enhance traditions that promote justice, especially fighting vices like discrimination, exclusion and stigmatisation of women and girls with disabilities in particular and persons with disabilities in general who find themselves in abject poverty. This resonates with biblical narratives as espoused by Jesus in the New Testament and some biblical prophets from the Old Testament, where believers are called upon to heed the cries of the less privileged and vulnerable members of society who are normally neglected by the contemporary global social, economic and political structures. The church, from within, has been able to focus on thematic issues that unite them in the fight against patriarchal and oppressive constructions of disability and sexuality. The church can be instrumental in deconstructing the societal purity myth and the naive hermeneutical disability theories that not only exclude the female body but also women with disabilities from participation in religious and cultural spaces. Ngezi Baptist Church is borrowing from the generic disability theology and the feminist disability theology, to be able to construct inclusive and accessible pastoral care ministry spaces that empower women and girls with disabilities. All these theologies conceive of God as contextual who must be reimagined in multiple diverse forms of human variation. This re-imagination of God can unlock the potential of the vulnerable categories and inspire the able-bodied and the differently abled persons to work together towards alleviating poverty. The theology is pro-life regardless of gender and disability and promises to be a potential source of inspiration for a combined effort between the church and government and other stakeholders in working out disability-friendly policies. The Ngezi Baptist Church is re-appropriating its theology of disability with a view to mitigating the stigmatisation, exclusion and discrimination of women and girls with disabilities and empowering them against poverty.
